# Lophomonas spp. as an Emerging Infectious Disease in the Caribbean

**DOI:** 10.7759/cureus.73510

**Published:** 2024-11-12

**Authors:** Rajeev P Nagassar, Peng Ewe, Shiva Jaggernauth, Sheena Boodoo, Stanley Giddings

**Affiliations:** 1 Microbiology, Sangre Grande Hospital, Sangre Grande, TTO; 2 Anaesthesiology, Southern Medical Clinic, San Fernando, TTO; 3 Internal Medicine, Southern Medical Clinic, San Fernando, TTO; 4 General Practice, Occupational Medical Providers, Couva, TTO; 5 Faculty of Clinical Medical Sciences, The University of the West Indies, St. Augustine, TTO

**Keywords:** caribbean, lophomonas blatarum, lophomonas spp, parabasalia, parabasalid, parasites, west indies

## Abstract

Over the past decade, there has been an increasing number of case reports of bronchopulmonary infection due to Lophomonas spp. The Caribbean has not been included in any reports. We describe two cases of bronchopulmonary infection due to *Lophomonas *spp. We also describe successful treatment with metronidazole and an alternative, albendazole. We also briefly review the controversy of *Lophomonas* spp. as a true pathogen and the population groups affected.

## Introduction

*Lophomonas blattarum* and its species are multi-flagellated anaerobic protozoan parasites [1]. *Lophomonas* spp. have been isolated from the guts of cockroaches and termites. These organisms excrete cysts in their fecal matter, and following cyst inhalation into the human respiratory tract, bronchopulmonary infection can occur. A recent systematic review of cases from 1993 to 2020 identified 307 cases [1]. These patients came from across 10 countries in four continents. The greatest number of reported cases were from Iran, followed by China. In South America, 22 cases were reported from Panama, Peru, and Mexico [1]. To date, there have been no reported cases of this emerging infectious disease from the Caribbean. We present two cases of bronchopulmonary infection due to *Lophomonas* spp., from two different islands, as we highlight an increase in the geographical range of this protozoan parasite.

## Case presentation

Case 1

A 71-year-old male with a history of hypertension and prior ischemic stroke with no residual deficit presented to the hospital after a cardiac arrest. Ventricular fibrillation was noted during his initial assessment. He received full cardiopulmonary resuscitation including defibrillation and there was a return of spontaneous circulation after approximately 30 minutes. He was intubated, placed on mechanical ventilation, and admitted to the intensive care unit. He was subsequently airlifted to another Caribbean Island five days later for a cardiology review.

On arrival at the intensive care unit at the tertiary care center, he was noted to have copious yellow endotracheal secretions. He had a low-grade temperature with a maximum temperature of 37.9°C. His blood pressure was 140/88 mmHg, and his heart rate was 82 beats/minute with regular rate and rhythm. He was on synchronized intermittent mandatory ventilation (SIMV) with a fraction of inspired oxygen (FiO_2_) of 50%, positive end-expiratory pressure (PEEP) of 10, respiratory rate of 16 breaths/minute, and oxygen saturation of 99%. The lung examination revealed bibasal crepitations and decreased air entry at the bases. He was sedated on propofol, and the rest of his examination was unremarkable.

**Figure 1:**
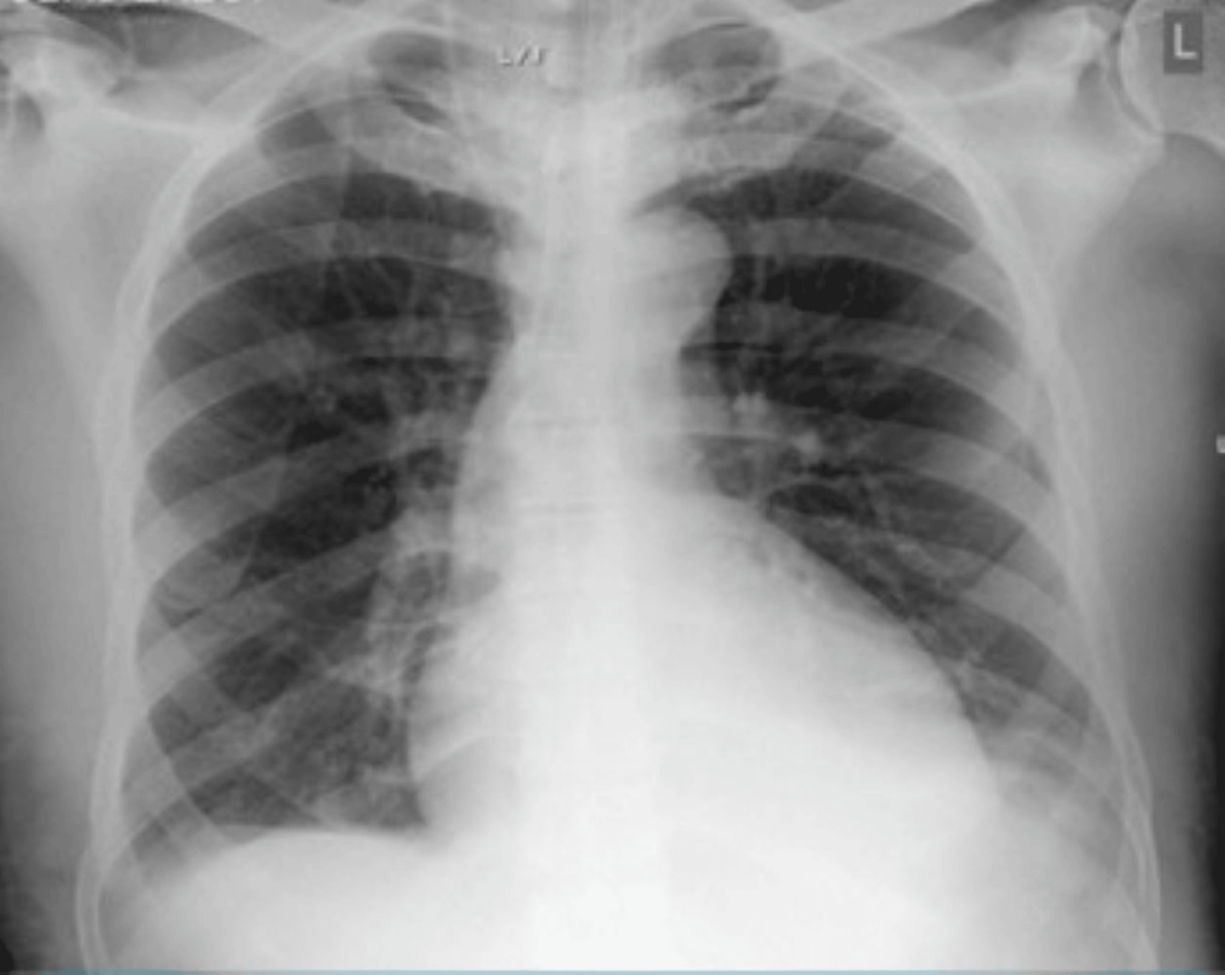
Chest X-ray of Case 1. This is the chest X-ray of Case 1 showing opacifications in the right mid to lower zones and opacification in the left mid-zone.

Endotracheal secretions were sent for culture, and he was placed on the broad-spectrum antibiotic piperacillin-tazobactam. A portable chest X-ray showed bilateral lower lobe infiltrates and consolidation. His complete blood count showed mild leukocytosis with a white blood cell count of 14 × 10^9^/L. There was mild eosinophilia on the differential. Endotracheal cultures subsequently returned negative. However, microbiology noted the presence of the protozoa in a bronchiolar lavage specimen, *Lophomonas* spp., was identified. Metronidazole was added to his regimen. His endotracheal secretions decreased over the next 48 hours, and he was subsequently extubated. Metronidazole was continued to complete a 14-day course of treatment, and his infection resolved. A repeat sputum sample one week later was negative for protozoa. (Table 1; Figure 1; Videos 1, 2).

**Table 1:** Complete blood count with differential and comprehensive metabolic panel for Case 1. This is the laboratory report of Case 1. It highlights elevated white blood cells and elevated eosinophils. Elevated eosinophils are usually seen in parasitic infections.

Tests performed	Result	Reference range
White cell count	14 × 10^9^/L	4.5–11.0 × 10^9^/L
Neutrophils	65.50%	40–75%
Lymphocytes	21.70%	20–50%
Monocytes	4.40%	2–10%
Eosinophils	6.80%	0–6%
Basophils	1.60%	0–3%
Haemoglobin	12.1 g/dL	14.0–17.5 g/dL
Platelet count	180 × 10^3^/µL	156–373 × 10^3^/µL
Serum potassium	3.5 mmol/L	3.5–5.1 mmol/L
Serum sodium	141 mmol/L	135–145 mmol/L
Serum creatinine	0.9 mg/dL	0.5–1.2 mg/dL
Blood urea nitrogen	16 mg/dL	3–20 mg/dL
Alanine aminotransferase	44 IU/L	20–60 IU/L
Aspartate aminotransferase	86 IU/L	5–40 IU/L
Total bilirubin	0.9 mg/dL	0.2–1.2 mg/dL
Alkaline phosphatase	53 U/L	40–129 IU/L
Albumin	3.5 g/dL	3.5–5.5 g/dL
Calcium	7.8 mg/dL	8.4–10.2 mg/dL
Phosphorus	3 mg/dL	2.3–4.7 mg/dL

**Video 1:** Lophomonas spp.: Case 1. The “to and fro” motion of the parasite is seen. The image has a high magnification for a better view of the movement.

**Video 2:** Lophomonas spp. of Case 1 with less magnification. This video of the *Lophomonas* spp. from Case 1 has less “zoom” and is clearer.

Case 2

A 78-year-old female with a history of chronic tobacco use had been experiencing symptoms of cough as well as dyspnea on exertion. She denied any fever or chills and there was no sputum production. Her pulmonologist sent her for a computerized tomography (CT) of the chest which showed a non-specific interstitial pattern with superimposed infection. Bronchoscopy and lavage were subsequently performed. The lavage fluid cultures as well as the acid-fast bacilli stain were negative. However, the microbiology lab noted the presence of *Lophomonas* spp. She was evaluated at the outpatient clinic. Her vital signs were normal. Lung auscultation was also normal.

**Figure 2:**
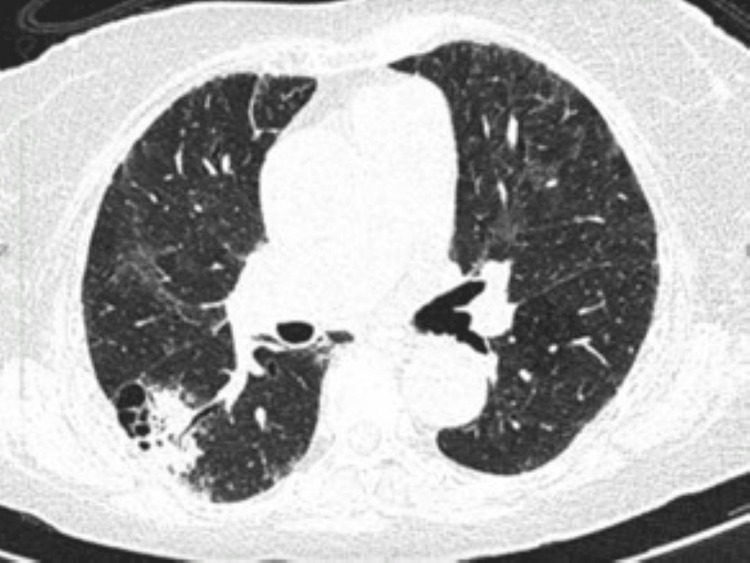
Computerized tomography (CT) of Case 2. A 4.2 cm macrocystic focus within the right lower lobe is associated with an area of opacification and air bronchograms, appearances are in keeping with a superadded infection.

**Figure 3:**
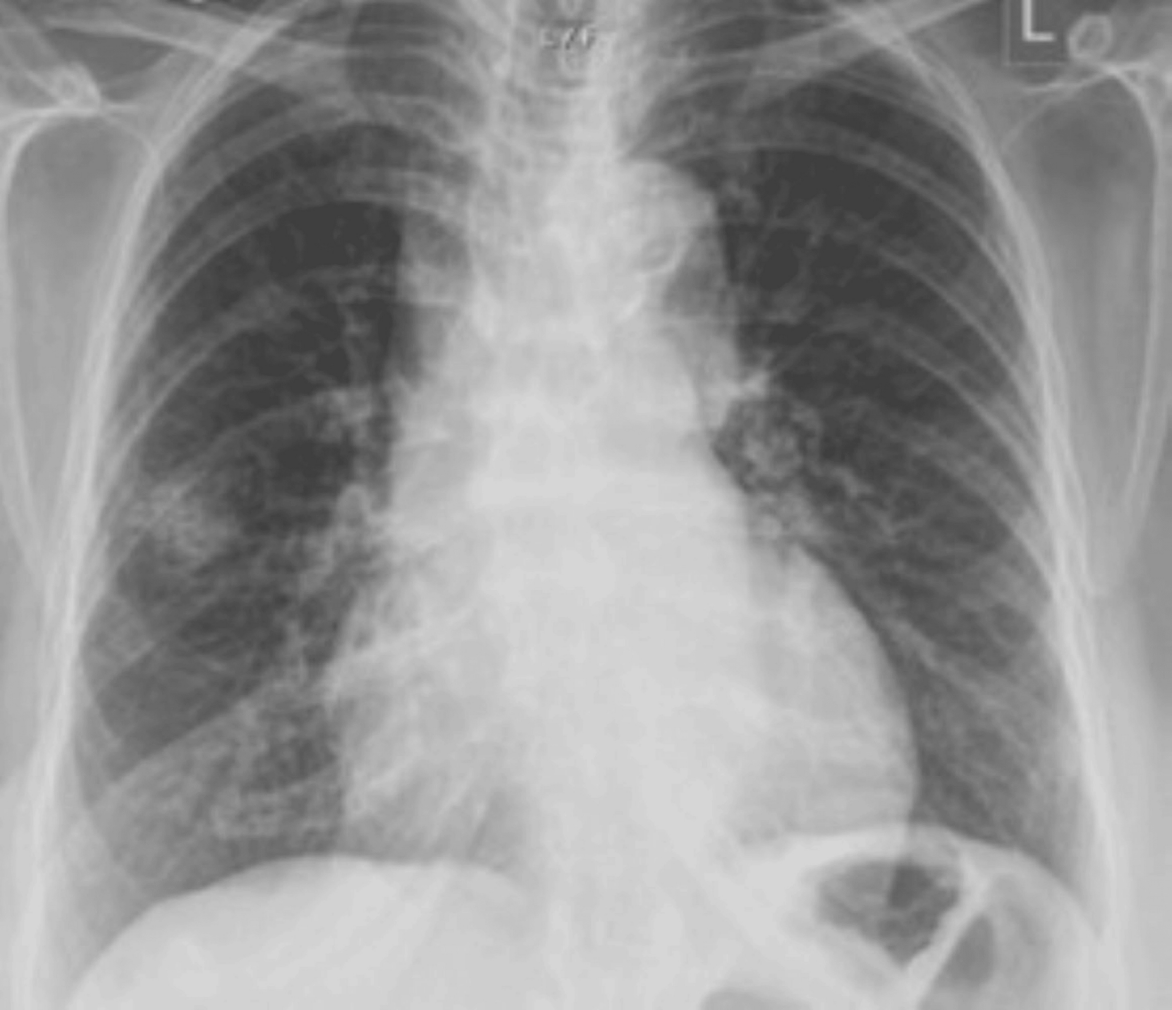
Chest X-ray of Case 2. A 2.2 x 1.3 cm focus of irregular opacification is seen within the right mid-zone.

**Figure 4:**
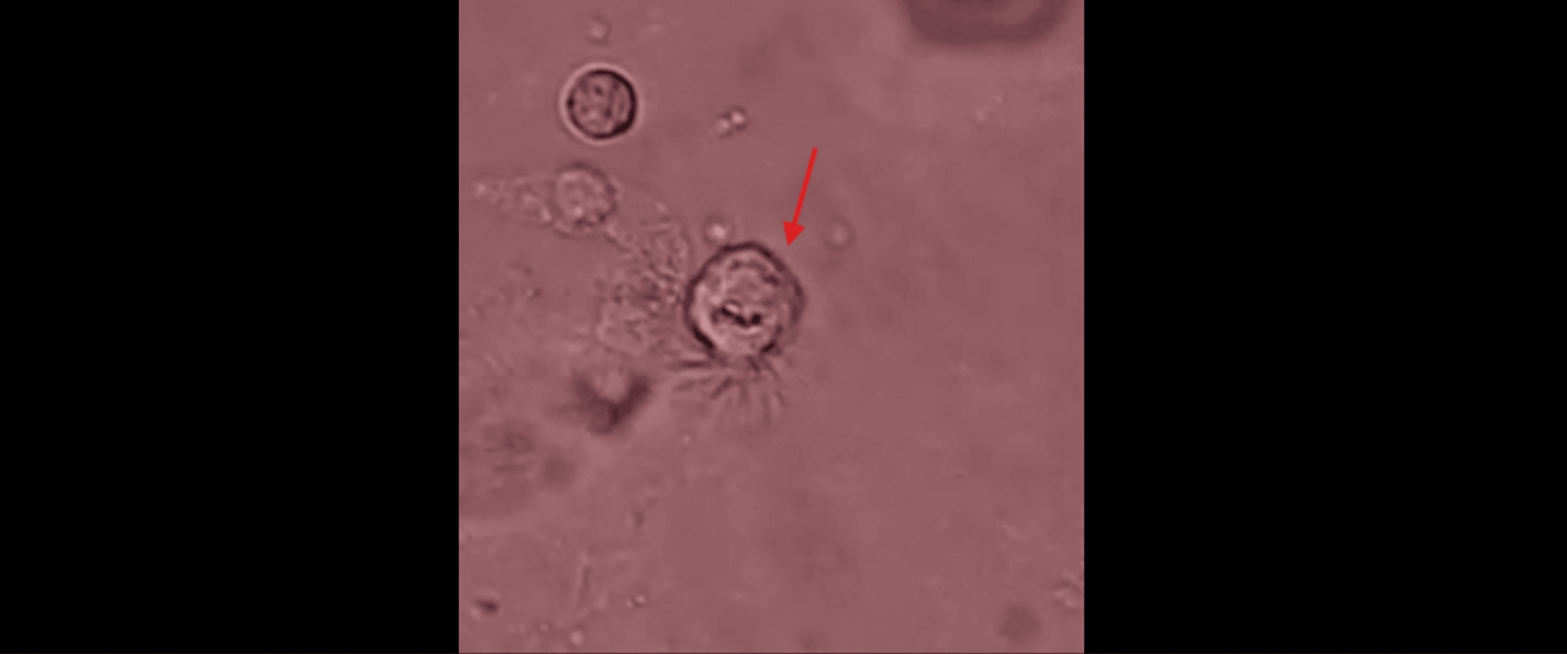
Lophomonas spp.: Case 2. The red arrow points to the parasite.

She was noted to be allergic to metronidazole. She was alternatively treated with albendazole 400 mg twice daily for five days. A sputum sample sent following treatment did not show any further protozoa. Her condition subsequently improved (Figures 2-4; Table 2; Video 3).

**Table 2:** Complete blood count and basic metabolic panel for Case 2.

Tests performed	Result	Reference range
White cell count	7.3 × 10^9^/L	4.5–11.0 × 10^9^/L
Neutrophils	62.60%	40–75%
Lymphocytes	28.10%	20–50%
Monocytes	6.10%	2–10%
Eosinophils	2.30%	0–6%
Basophils	0.90%	0–3%
Haemoglobin	15.4 g/dL	11.5–16.5 g/dL
Platelet count	336 × 10^3^/µL	156–373 × 10^3^/µL
Serum potassium	4.6 mmol/L	3.5–5.1 mmol/L
Serum sodium	141 mmol/L	135–145 mmol/L
Serum creatinine	0.6 mg/dL	0.5–1.2 mg/dL
Blood urea nitrogen	7 mg/dL	3–20 mg/dL
Fasting blood sugar	94 mg/dL	60–120 mg/dL

**Video 3:** Lophomonas spp.: Case 2. The “to and fro” motion of the parasite is seen. The cilia, round to piriform shape, and nucleus are seen.

## Discussion

*Lophomonas* spp. are flagellated protozoan parasites that belong to the phylum Parabasalia [1-3]. A rise in the number of cases or increased geographical range confers this protozoa, *Lophomonas *spp., as an emerging infectious disease [1]. Several case reports of bronchopulmonary infection have been identified across various geographical locations around the world [2]. Cases have been reported in both immunocompromised and immunocompetent patients as well as pediatric and adult patients. Both of our patients were elderly adult patients who presented with respiratory symptoms [1,4]. Clinical symptoms of patients reported in the literature have been non-specific. One of our patients had a low-grade temperature while the other patient was afebrile. One patient had increased respiratory secretions while the other patient required bronchoscopy and lavage to obtain a respiratory sample. Laboratory and radiological findings also differed in our patients [4]. This case presentation contradicts the finding of Nakhaei et al., who reported that this may be mainly affecting young, healthy patients, as our patients were elderly [1].

Diagnosis of *Lophomonas* spp. requires identification of the organism in respiratory samples [4]. Given the non-specific nature of symptoms, it is unlikely that the clinician will alert the microbiologist that they suspect this organism. Microbiologists must consider this organism on their differential once they identify multi-flagellated organisms on examination of respiratory samples. The organism can be mistaken for bronchial ciliated epithelial cells. However, several distinguishing factors can be used to differentiate this parasite such as the “to and fro” motion, pyriform or spherical shape, tufts of flagella, and a nucleus located at the base of the flagella tufts [4,5]. Although the author of “Is this a Parasite or is it Just Me?” questions the validity of the “to and fro” motion, this was clearly seen in the specimens from our cases. In fact, the organisms were not shaped like ciliated epithelial cells either. Other resources are available to assist with diagnosis and centers have developed in-house molecular techniques; however, our center did not have molecular detection capability [6]. Our cases were confirmed by the identification of the “to and fro” movement of the protozoa, tufts of flagella, the nucleus, and characteristic spherical to piriform appearances.

Notably, authors such as Mewara et al. have questioned the validity of concluding that infections have been caused by *Lophomonas *spp. The authors concluded that ciliated epithelial cells were misidentified as *Lophomonas* spp. They further stated that the pictures and videography from human specimens were not representative of true *Lophomonas* spp. infection. Additionally, the authors noted the dearth of clinical evidence [6]. Nakhaei et al., in their systematic review of *Lophomonas* cases, reviewed 32 eligible articles. They found that most cases belonged to the younger age group, unlike our cases. However, similar to our cases, bronchiolar lavage was utilized in most cases. This study concluded that the number of *Lophomonas* cases may be underestimated, unlike the conclusion of Mewara et al. [1,6]. Thus, the literature is conflicting with regard to this emerging pathogen. This adds to the importance of our two cases presented here. Kilimcioglu et al. examined the presence of flagellated protozoa parasites in the bronchoalveolar lavage specimens of immunocompromised patients and found that in a small percentage, the symptoms could be attributed to flagellated organisms such as *Lophomonas* spp. [7]. das Neves Coelho et al. recently reported a small number of cases from tracheal aspirates or bronchoalveolar lavage from intensive care unit patients adding to the narrative that the infection of the lung by *Lophomonas* spp. can occur in immunocompromised individual [8]. Clinical features, radiographs, and laboratory findings were utilized, as in our cases. In addition, das Neves Coelho et al. used an expert parasitologist; in our case, we utilized an expert microbiologist who looked at similar features of the parasite such as the shape of the organism, nucleus, cilia, and movement. In our case, we describe a “to and fro” movement [8].

Metronidazole is the treatment of choice for the protozoa [2]. According to Martinez-Girón et al., the dosage is 500 mg every eight hours orally for 7-10 days in adults. This was considered as an option. Our male patient was successfully treated with metronidazole and his symptoms improved. However, our female patient reported an allergy to metronidazole. She was treated with albendazole, and her condition improved [9]. As more cases are identified in the Caribbean, we may encounter issues such as our second patient who had a metronidazole allergy.

As the number of cases and geographical expansion of *Lophomonas* spp. occurs, especially due to increasing immunocompromised populations such as the elderly, clinicians need to be aware of this emerging infectious disease. Informed identification, diagnosis, and treatment are key to resolving this infection [1-3,7]. This strengthens the importance of the finding of these two cases in a new part of the world.

## Conclusions

There are differing opinions in the literature about* Lophomonas* spp. as a true pathogen. We present two cases of true infection in the elderly, which were successfully treated in the Caribbean. Lastly, we give an alternative to albendazole for people allergic to metronidazole.
